# Identification of Important Genes Involved in the Sex-Differentiation Mechanism of Oriental River Prawn, *Macrobrachium nipponense*, During the Gonad Differentiation and Development Period

**DOI:** 10.3389/fgene.2022.797796

**Published:** 2022-02-15

**Authors:** Shubo Jin, Wenyi Zhang, Yiwei Xiong, Sufei Jiang, Hui Qiao, Yongsheng Gong, Yan Wu, Hongtuo Fu

**Affiliations:** Key Laboratory of Freshwater Fisheries and Germplasm Resources Utilization, Ministry of Agriculture, Freshwater Fisheries Research Center, Chinese Academy of Fishery Sciences, Wuxi, China

**Keywords:** *Macrobrachium nipponense*, gonad differentiation and development, reproduction-related genes, regulation of gonad development, transcriptome profiling analysis

## Abstract

Identification of important genes, involved in the gonad differentiation and development, plays essential roles in the establishment of the artificial technique to regulate the process of testis development in *M. nipponense*. In this study, we aimed to determine the sensitive period of gonad differentiation and development through hematoxylin and eosin (HE) staining. The important genes involved in the gonad differentiation and development of *M. nipponense* were then identified through transcriptome profiling analysis during the sensitive period of gonad differentiation and development. HE staining analysis revealed that the sensitive period of gonad differentiation and development was from the post–larval developmental stages 5 (PL5) to PL25, which was dramatically faster than was for the other identified aquatic animals. The transcriptome profiling analysis predicted that phagosome, lysosome, oxidative phosphorylation, and glycolysis/gluconeogenesis play essential roles in the mechanism of gonad differentiation and development in *M. nipponense*. A total of 29 genes were further identified as the candidate genes, involved in the process of gonad differentiation and development in *M. nipponense*, based on the gene annotation and gene expression pattern. The qPCR analysis of *Mn-JHEH*, *Mn-DHP*, *Mn-ALY*, and *Mn-SMA6* during the whole developmental process revealed that all of these four genes showed high expression levels during the sensitive period of gonad differentiation and development in *M. nipponense*. *Mn-JHEH*, *Mn-DHP*, and *Mn-ALY* showed higher expressions at PL25F than at PL25M, while *Mn-SMA6* showed a higher expression at PL25M. The RNA interference (RNAi) analysis was further used to investigate the potential functions of *SMA6* in male sexual development of *M. nipponense*. The RNAi analysis revealed that *SMA6* positively regulated the testis development in *M. nipponense* by affecting the expression of *Mn-IAG*. This study provided valuable evidences for the establishment of the technique to regulate the process of gonad development in *M. nipponense*.

## Introduction

The oriental river prawn, *Macrobrachium nipponense* (Crustacea, Decapoda, Palaemonidae), is an important commercial species in China (Fu et al., 2012). The annual aquaculture production reached to 225,321 tons in 2019, producing huge economic benefits (Zhang et al., 2020). The male prawns of *M. nipponense* grow faster and reach larger sizes at the harvest time than the female prawns, which is consistent with other *Macrobrachium* species. Thus, the male prawns are preferred in *M*. *nipponense* aquaculture (Fu et al., 2012). Rapid gonad development has negative effects on the sustainable development of the *M. nipponense* aquaculture industry. Both testes and ovaries can reach sexual maturity within 40 days after hatching in *M*. *nipponense* aquaculture. This results in inbreeding, leading to multiple generations in the same pond and the degradation of germplasm quality, smaller size, and decreased ability to resist diseases (Jin et al., 2021). Therefore, it is of urgent need to fully understand the gonad differentiation and development mechanism in *M. nipponense*. These play essential roles in the establishment of the technique to produce all male progeny on a commercial scale and to regulate the process of gonad development in *M*. *nipponense*.

The androgenic gland is a specific gland found in most crustaceans. The hormones secreted by the androgenic gland play crucial roles in driving the male sexual differentiation and development, especially the development of the testes (Sagi et al., 1986; Sagi et al., 1990). In *Macrobrachium rosenbergii*, the ablation of the androgenic gland from the male prawns resulted in sex reversal to the female phenotype (Sagi et al., 1986; Sagi et al., 1990). Insulin-like androgenic gland hormone (*IAG*) is the main focused gene, produced by the androgenic gland (Ventura et al., 2009; Rosen et al., 2010; Ventura et al., 2011). It has been proven to play essential roles in promoting male sex-determination and sex-differentiation in most crustacean species, including *Fenneropenaeus chinensis* (Li et al., 2012), *Scylla paramamosain* (Huang et al., 2014), Hermaphrodite Shrimp (Liu et al., 2021), *Fenneropenaeus merguiensis* (Zhou et al., 2021), and *M. nipponense* (Li et al., 2015). Silencing of *IAG* in male *M. rosenbergii* by RNA interference may also lead to complete sex reversal (Ventura et al., 2012). A previous study reported that *IAG* was specially expressed in the androgenic gland (Ma et al., 2016).

The abovementioned studies have received a lot of attention on the mechanism of sex differentiation and development in *M. nipponense*. Some transcriptome analyses have been conducted in *M. nipponense* based on the testis and androgenic gland, and a series of genes were identified from these transcriptome analyses (Jin et al., 2013; Jin et al., 2015; Jin^A^ et al., 2018), which have been proven to be involved in the mechanism of male sexual development (Jin et al., 2014; Jin^B^ et al., 2018; Jin et al., 2019; Jin^A^ et al., 2021; Zhang et al., 2013). Identifications of the sensitive period of gonad differentiation and development have been performed in many aquatic species. The sensitive period of gonad differentiation and development is from the beginning of gonad differentiation to the formation of sperms and oocytes. The sensitive period of testis differentiation of *Larimichthys crocea* was from 95 days after hatching, and spermatocyte and lobules were observed at 215 and 230 days after hatching, respectively. The sensitive period of ovary differentiation was from 60 days, and oocytes were observed at 120 days after hatching (You et al., 2012). The sensitive period of testis and ovary differentiation was observed at 30 and 25 days after hatching in *Misgurnus anguillicaudatus*, respectively (Chen et al., 2007). The sensitive period of testis and ovary differentiation of *Takifugu obscurus* was observed at 40 and 35 days after hatching, respectively (Dai, 2010). The testis differentiation of *Pseudobagrus fulvidraco* was observed from 60 days after hatching, and the ovarian differentiation was observed earlier to the testis differentiation (You et al., 2016). The sensitive period of testis and ovary differentiation was observed at 90 and 80 days after hatching in *Varicorhinus macrolepis*, and their maturation needed 36 and 24 months, respectively (Song et al., 2006). However, the studies related to the identification of important genes during the gonad differentiation and development period have been rarely reported. Identification of the genes during the gonad differentiation and developmental period may dramatically promote the understanding of the sex-differentiation mechanism in *M. nipponense*, advancing the researches to regulate the process of gonad development in *M*. *nipponense*.

In this study, the gonad-differentiation sensitive period was determined by using the histological observations, including the formation of the testis and ovary. The transcriptome profiling analysis was also performed during the gonad differentiation and development period in order to identify the candidate metabolic pathways and genes involved in the mechanism of gonad differentiation and development in *M. nipponense*. This study provides valuable evidence for the studies on the sexual differentiation and development in *M. nipponense*, as well as for the whole of the crustacean species.

## Materials and Methods

### Ethics Statement

Permission was obtained from the Tai Lake Fishery Management Council and the committee of Freshwater Fisheries Research Center during the experimental programs. All experiments were performed in accordance with relevant guidelines and regulations.

### Sample Collection

A total of 50 healthy pregnant female prawns were collected from a wild population in Tai Lake in July, Wuxi, China (120°13′44″E, 31°28′22″N) and maintained in lab conditions, with the room temperature at 28°C and dissolved oxygen at ≥6 mg/L. The specimens for the histological observations were collected from the post–larval development stages PL1 (Day 1) to PL30, and samples were collected every 5 days (*n* = 5). The male and female prawns can be visually distinguished at PL25. A total of five specimens at PL5, PL10, PL15, PL25M (male prawns at PL25), and PL25F (female prawns at PL25) were respectively collected for the transcriptome profiling analysis to form a biological replicate, and three replicates were performed based on the analysis of gonad differentiation and development of the sensitive period. The specimens for the whole developmental process were collected for qPCR analysis, including the embryonic developmental stages, larval developmental stages, and post–larval developmental stages. The samples for the embryonic developmental stages were collected according to the description provided in a previous study (Chen et al., 2012). The full-sibs population was hatched and cultured, and specimens at different larval and post-larval developmental stages were collected every five days during their maturation process. (*n* = 5). Another 10 male and female prawns were collected from the Tai Lake in July 2019 and maintained in lab condition with the room temperature at 28°C and dissolved oxygen at ≥6 mg/L for 72 h prior to the tissues collection. The collected mature tissues included the eyestalk, brain, heart, hepatopancreas, gills, muscles, and gonads (*n* = 5), and each of the tissues was respectively collected from both the male and female prawns for qPCR analysis. The tissue samples were immediately frozen in liquid nitrogen and stored at −80°C until RNA extraction, in order to prevent RNA degradation.

### Histological Observations

The sensitive period of gonad differentiation and development of *M. nipponense* was determined by hematoxylin and eosin (HE) staining. The detailed procedures for HE staining have been provided in previous studies (ShangGuan et al., 1991; Ma et al., 2006). The histological slides were observed under the Olympus SZX16 microscope (Olympus Corporation, Tokyo, Japan). The cell types of the testes and ovaries were observed and labelled based on previous studies (Jin^A^ et al., 2021; Li et al., 2018).

### Transcriptome Profiling Analysis

The differentially expressed genes during the sensitive period of gonad differentiation and development of *M. nipponense* were identified through the transcriptome profiling analysis. The transcriptome profiling analysis was performed on an Illumina High-seq 2500 sequencing platform. Previously published studies have described well the detailed procedures for RNA-Seq and analysis (Jin et al., 2013; Jin^B^ et al., 2021). The Trinity program (version: trinityrnaseq_r20131110) was used to assemble the clean data into nonredundant (Nr) transcripts (Grabherr et al., 2011). Gene annotation was then performed in the Nr database, and the Gene Ontology (GO) (Ashburner et al., 2000), Cluster of Orthologous Groups (COG) (Tatusov et al., 2003), and Kyoto Encyclopedia of Genes and Genomes (KEGG) databases (Minoru et al., 2008), using an E-value of 10^−5^ (Jin et al., 2013). The EBSeq algorithm was used to filter the differentially expressed genes, under the criteria of the false discovery rate being <0.05 (Benjamini et al., 2001).

### qPCR Analysis

qPCR was used to verify the accuracy of RNA-Seq and determine the mRNA expression of important differentially expressed genes (DEGs) during the whole developmental process. The detailed procedures have been well described in previously published studies (Jin^B^ et al., 2018; Jin et al., 2019; Zhang et al., 2013). The qPCR analysis was performed on the Bio-Rad iCycler iQ5 Real-Time PCR System (Bio-Rad), and the SYBR Green RT-qPCR assay was used. The primers for the qPCR analysis are listed in Table 1. The eukaryotic translation initiation factor 5A (*EIF*) was used as a reference gene in this study (Hu et al., 2018). The relative expression levels were measured by using the 2^−ΔΔCT^ method (Livak and Schmittgen, 2001).

**TABLE 1:** Primers used in this study.

Name	Sequence
Hemocyanin subunit 1	F: TAT​GCC​AAA​ACC​TTC​GAT​CCA​GA
	R: TCT​TCT​CCA​AAG​TAG​GCA​GCA​TT
Chitinase 4	F: GGA​TTG​AAG​TCT​TTC​AGT​GCG​AC
	R: TCA​CCT​GTG​TAC​TCA​CCT​GAT​CT
Hemoglobin	F: TCT​TCT​GTC​ACT​GTT​CCT​GAT​GT
	R: GCC​AGG​ATG​AGA​GAA​AAG​TCC​AT
*JHEH*	F: TGG​AGT​TTT​ACA​GCA​TTT​TGC​CG
	R: ATG​TTT​CCA​GAA​GTC​CAA​GAC​CT
Hemocyanin	F: ACT​GGT​TCT​CCC​TTT​TCA​ACG​AA
	R: AAA​GGG​CAT​ACA​CAA​ACT​CTC​CT
GH16146	F: TAC​TAC​CAC​GGG​GAG​ACC​ATT​AT
	R: GTA​CTT​GTT​GTG​GTT​GGT​GGA​AG
Dihydropyrimidinase	F: CAC​AAG​AGA​TGG​AGG​AAC​TCA​CA
	R: CCT​CTT​CAG​GAC​GAC​TCA​TTT​CA
Ubiquitin-2	F: CCC​TTA​CAG​GAA​AGA​CAA​TCA​CC
	R: TAA​GGG​TCT​TCA​CAA​AGA​TCT​GCA​T
*SMA6*	F: AAT​CAC​TGC​TCT​TTC​TCC​ATC​CA
	R: CTC​ATC​GTA​CTC​TTC​CTT​GGT​GA
Kynurenine formamidase	F: ACG​TCT​CGA​AGA​ATA​TCA​AGC​CA
	R: TGT​ATT​GAC​CTT​AGC​ACC​CCA​TC
Hemocyanin subunit 2	F: CAA​TGG​AGT​GTA​CCC​TGA​CAA​GA
	R: ATG​GGA​ATG​ATG​GAA​GTT​GGT​GA
LOC580379	F: AGT​CTC​GTT​GAA​TCC​TTG​TCT​CC
	R: TTT​ACA​GCA​CGA​TAA​CGG​AAG​GA
Hypothetical protein	F: GAA​TGA​AAG​GTT​GGA​CGA​GGG​AT
	R: TCC​ACA​AAG​AAG​GAT​CAC​ACA​CT
*ALY*	F: AAG​GTC​CCC​AAC​AAC​AAA​TAG​GA
	R: GCT​GTT​GTG​GCA​TTG​TTT​TCT​TC
beta-N-acetylglucosaminidase	F: CCA​ACC​CAA​AGA​TGT​ATG​ACG​TG
	R: AGA​GAG​GCC​GTA​GTC​ATT​TTC​AA
NADH-cytochrome b5 reductase	F: CAA​TCT​GGC​TCT​TCT​CAT​AGC​CT
	R: TTC​GCT​GTG​TCT​TGA​ATT​TTC​CC
Hypothetical protein	F: ACG​ATT​GGC​ATG​GAG​TCT​GTA​AT
	R: TCA​AAG​TGA​TGG​CAG​TAT​TGG​GA
DAPPUDRAFT_92219	F: AGG​TTT​CGA​TGG​TCC​TGT​CAT​AG
	R: CCG​ATG​ATC​TTG​TGG​CTA​TCA​GT
Oplophorus-luciferin 2	F: GCT​GAA​AAG​AAT​ACG​AGA​CCA​CG
	R: GGA​CTT​TCT​TCT​GGA​CCA​AAT​GC
EIF	F: GTT​GTA​TGC​AGT​CGG​CCA​TAT​TT
	R: TGT​CCT​GAA​GGT​GGT​GAT​AAT​GA
IAG	F: CGC​CTC​CGT​CTG​CCT​GAG​ATA​C
	R: CCT​CCT​CCT​CCA​CCT​TCA​ATG​C
SMA6-RNAi	F:TAATACGACTCACTATAGGGAGGTTTCGATGGTCCTGTCATAG
	R:TAATACGACTCACTATAGGGCCGATGATCTTGTGGCTATCAGT

### RNA Interference Analysis

RNA interference (RNAi) was performed to analyze the potential functions of *SMA6* in the mechanism of male sexual development in *M*. *nipponense*. The specific RNAi primer with T7 promoter site was designed in the open reading frame of *Mn-SMA6*, using the Snap Dragon tools (http://www.flyrnai.org/cgibin/RNAifind_primers.pl) (Table 1). The *Mn-SMA6* dsRNA was synthesized by the Transcript Aid™ T7 High Yield Transcription kit (Fermentas, Inc., United States) based on the manufacturer's protocol. About 300 healthy *M*. *nipponense* male prawns with a body weight of 1.85–2.74 g were collected from the Tai Lake in Wuxi, China (120°13′44″E, 31°28′22″N). These male prawns were randomly divided into the RNAi and control groups, and each group has 150 prawns. As described in a previous study (Jiang et al., 2014; Li et al., 2018), prawns from the RNAi group were injected with 4 μg/g *Mn-SMA6* dsRNA. Thus, the concentration of the *Mn-SMA6* dsRNA was diluted to 4 μg/μL, and the content of the injected *Mn-SMA6* dsRNA was based on the prawn's body weight. The prawns from the control group were injected with an equal volume of green fluorescent protein (*GFP*), according to the prawn's body weight. The androgenic glands were collected from both the control and RNAi groups at Days 1, 7, and 14 after *GFP* and *Mn-SMA6* dsRNA injection and the *Mn-SMA6* mRNA expression by qPCR was measured, permitting confirmation of the silencing efficiency (*n* ≥ 5). The mRNA expression of *Mn-IAG* was also measured in the same cDNA templates in order to analyze the regulatory relationship between *Mn-SMA6* and *Mn-IAG*.

### Statistical Analysis

The quantitative data are expressed as mean ± SD. The statistical differences were estimated by one-way ANOVA followed by LSD and Duncan's multiple range test. All statistical analyses were performed using SPSS Statistics 23.0. A probability level of 0.05 was used to indicate significance (*p* < 0.05).

## Result

### Histological Observations of Gonad Differentiation and Development

**FIGURE 1:**
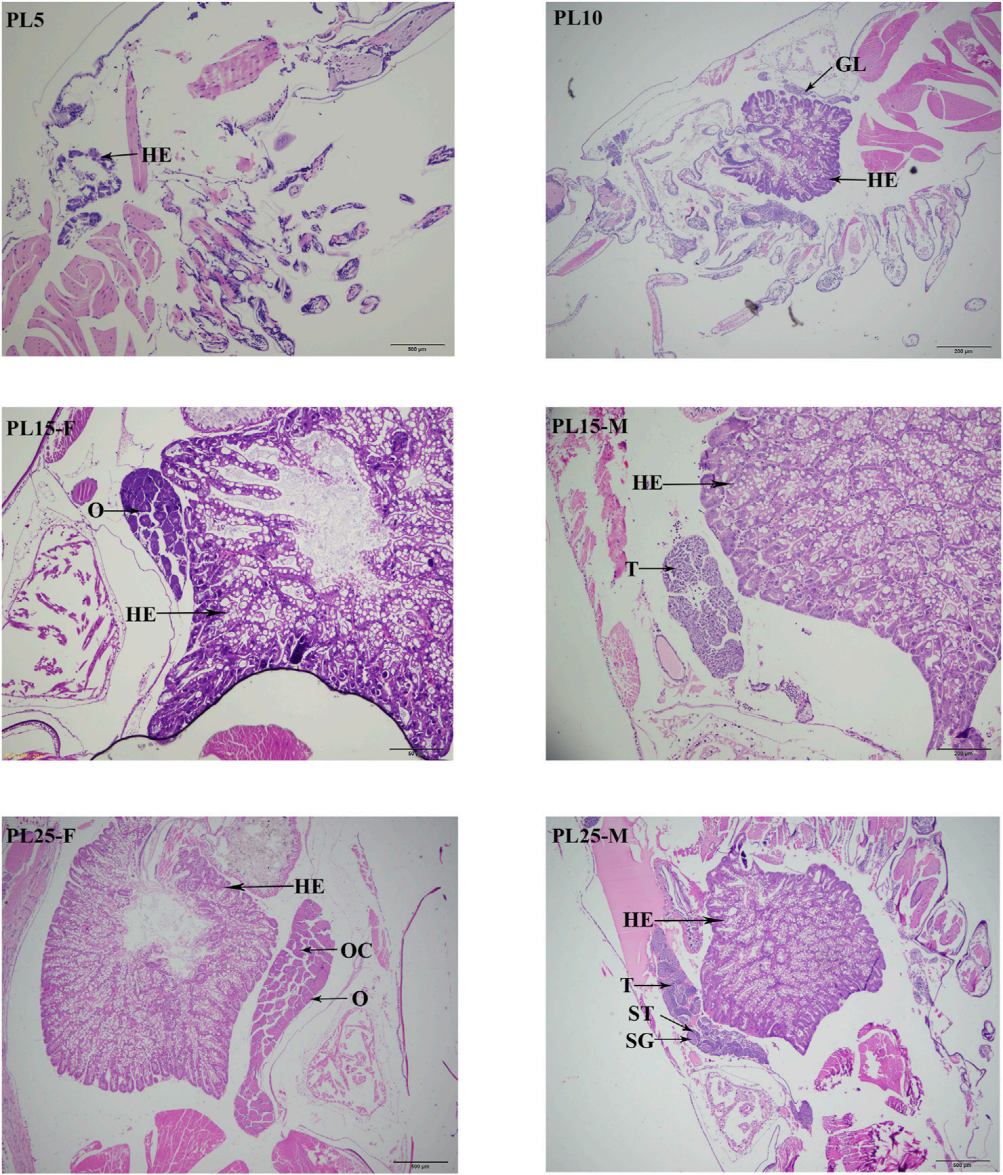
Identification of sensitive period of gonad differentiation and development in juvenile prawns of *M. nipponense*. HE: hepatopancreas; GL: germ gland anlage; OC: oocyte; SG: spermatogonia; ST: seminiferous tubules; O: ovary; T: testis. Scale bars = 20 μm.

According to the histological observations (Figure 1), the gonadal primordia was not observed at PL5, while the gonadal primordia was observed at the PL10 for the first time. The testes and ovaries were differentiated and observed for the first time at the PL15. However, the sex cannot be visually distinguished at this period. The sex can be visually distinguished for the first time at PL25. The sperms can be observed in the testes at PL25, and the ovaries had developed to stage Ⅳ.

### Transcriptome Analysis

A total of 49,103 Nr transcripts were assembled in this transcriptome with an average length of 1279.31 bp. The main transcripts ranged from 301 to 400 bp (22.42%) in length, followed by >2000 bp (20.53%) and 401–500 bp (12.87%). A total of 18,430 unigenes (37.53%) were matched to known proteins in the Nr database. The other unannotated unigenes represent novel genes, and the functions need further investigations.

**FIGURE 2:**
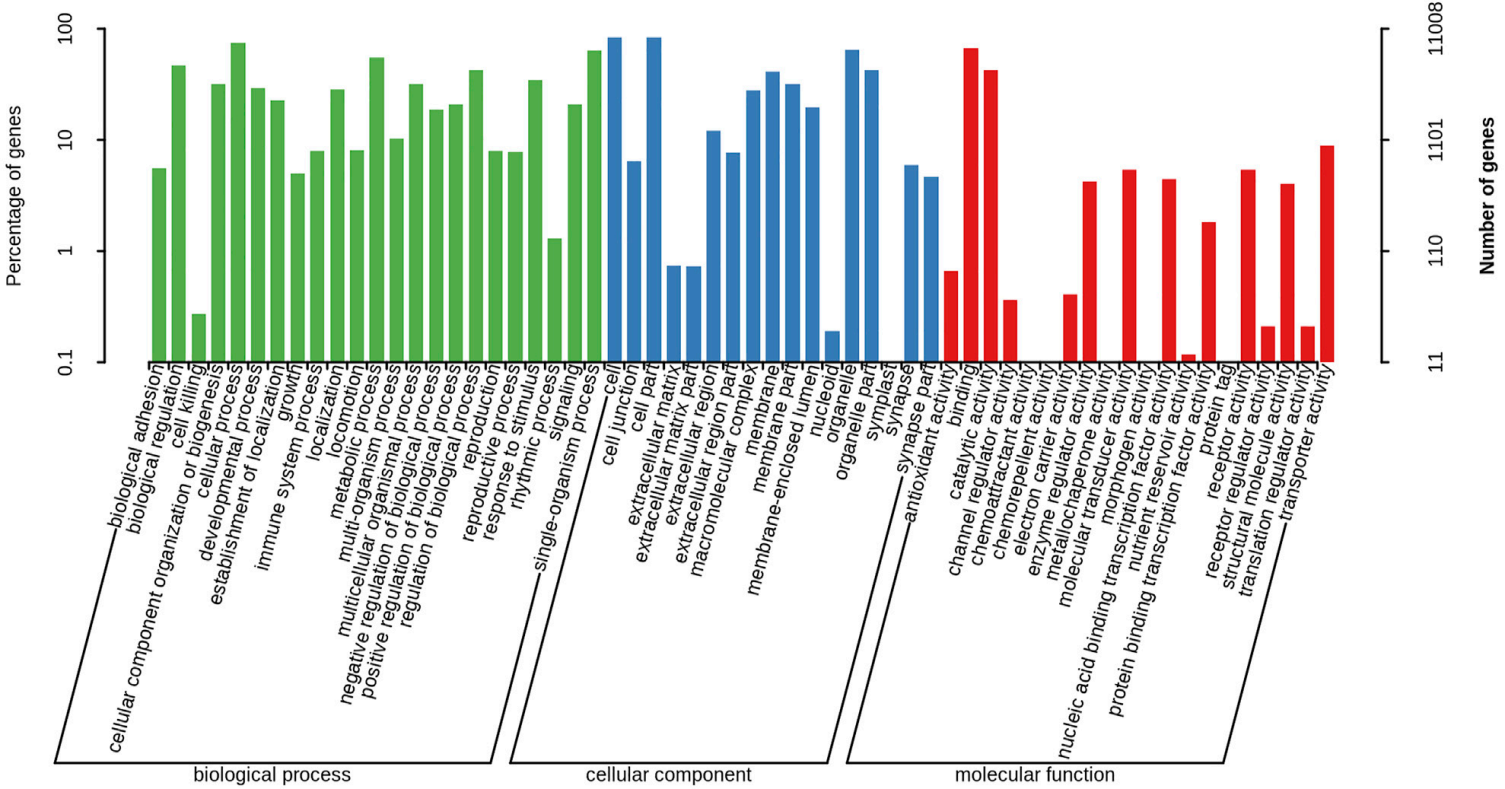
Gene Ontology classification of nonredundant transcripts. The left *y*-axis and right *y*-axis indicate the percentage and the number of a specific category of genes existed in the main category, respectively.

**FIGURE 3:**
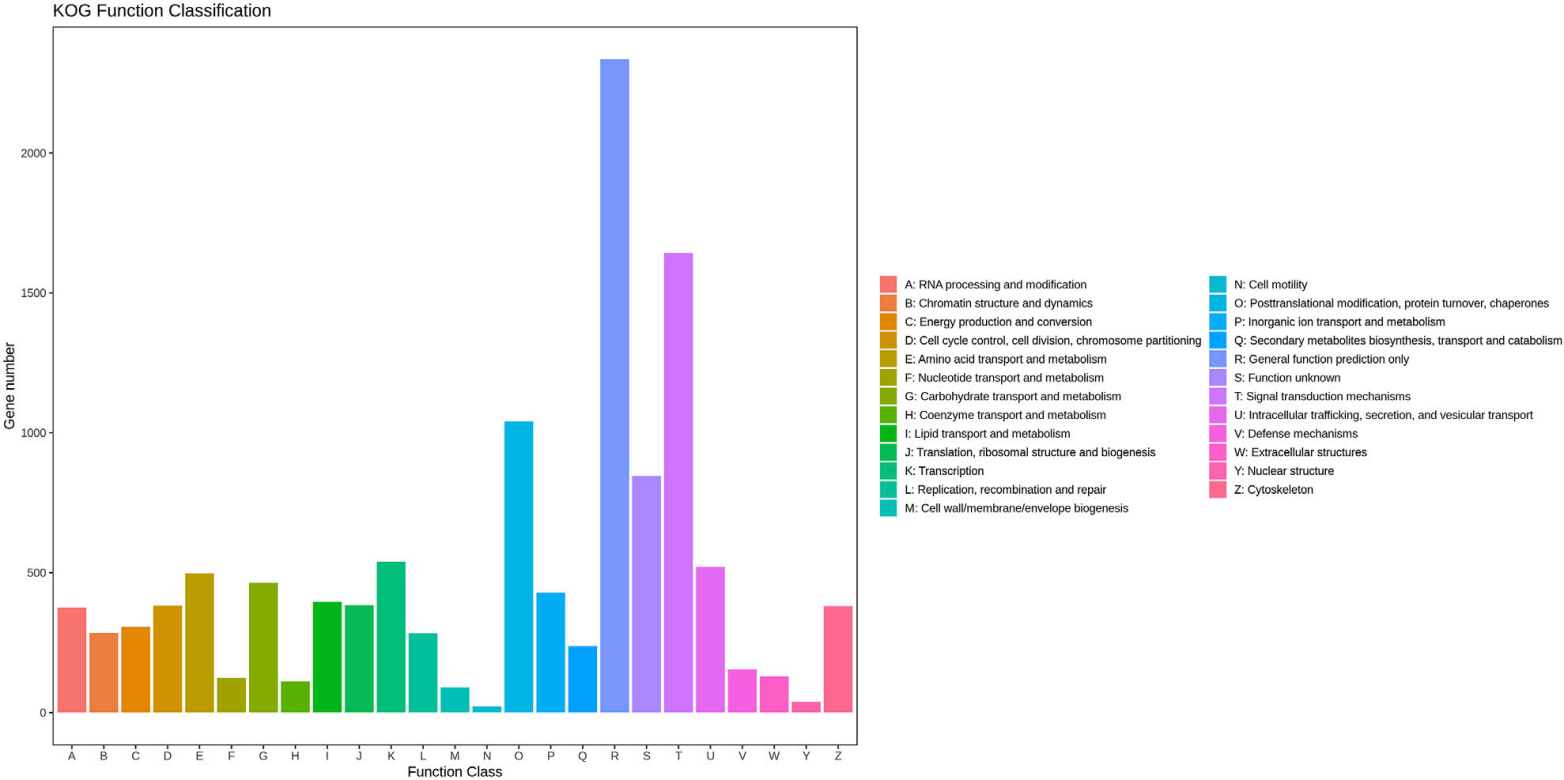
Cluster of orthologous groups (COG) classification of putative proteins.

A total of 11,008 unigenes (22.42%) and 10,613 unigenes (21.61%) were annotated in the GO and COG databases, respectively. The functions of the transcripts can be vocabular described by the GO and COG analysis. The GO analysis mainly included three categories with 60 functional groups: biological processes (9,444 unigenes, 23 functional groups), cellular components (10,136 unigenes, 17 functional groups), and molecular functions (9,563 unigenes, 20 functional groups) (Figure 2). The number of annotated transcripts in each functional group ranged from 1 to 9,181. The main functional groups in the GO analysis included cell, cell part, cellular process, binding, and organelle, in which the number of unigenes were more than 7,000. The COG analysis comprised of 25 functional categories, and the number of unigenes in each functional category ranged from 2 to 2,335 (Figure 3). The general function prediction only, signal transduction mechanisms, and post-translational modification, protein turnover, chaperones, in which the number of unigenes were >1000 represented the main functional categories.

A total of 4,980 unigenes (10.14%) were annotated in the KEGG database, which participated in the 340 metabolic pathways. The metabolic pathways, in which the number of unigenes were more than 200 included pathways in cancer, Alzheimer disease, Huntington disease, and lysosomes.

### Identification of Differentially Expressed Genes

**FIGURE 4:**
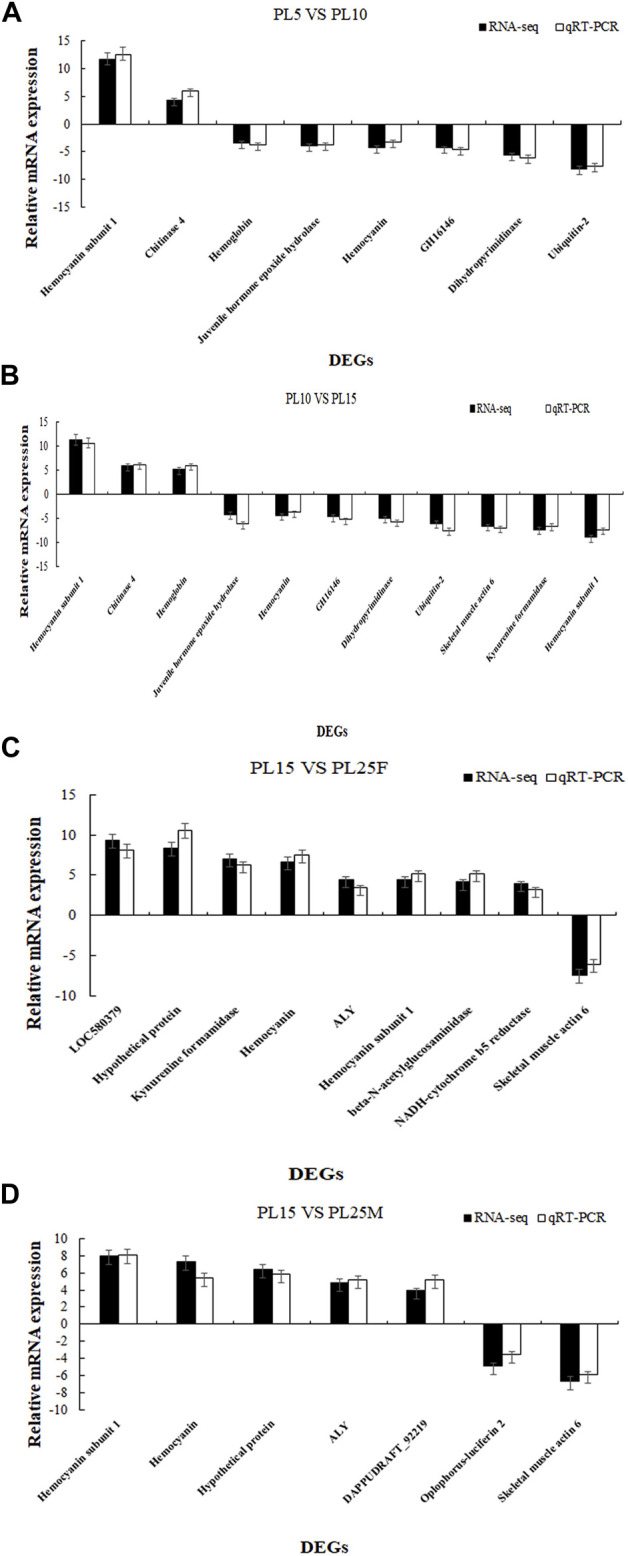
**(A)** Verification of DEGs between PL5 vs. PL10; **(B)** verification of DEGs between PL10 vs. PL15; **(C)** verification of DEGs between PL15 vs. PL25F; and **(D)** verification of DEGs between PL15 vs. PL25M.

The differentially expressed genes were identified, using the criteria of >2.0 as upregulated genes and <0.5 as downregulated genes and the *p*-value of <0.05. The number of DEGs between PL5 and PL10 was 482, including 340 upregulated genes and 142 downregulated genes. A total of 98 DEGs were identified between PL10 and PL15, including 20 upregulated genes and 78 downregulated genes. A total of 240 and 208 DEGs were identified between PL15 and PL25F (female prawns at PL25), and PL15 and PL25M (male prawns at PL25), respectively. The DEGs between PL15 and PL25F included 187 upregulated genes and 53 downregulated genes. The DEGs between PL15 and PL25M included 126 upregulated genes and 82 downregulated genes. The phagosome, lysosome, oxidative phosphorylation, and glycolysis/gluconeogenesis were the main enriched metabolic pathways between PL10 *vs.* PL15, PL15 *vs.* PL25F, and PL15 *vs.* PL25M, while the adrenergic signaling in cardiomyocytes, cardiac muscle contraction, starch and sucrose metabolism, and glycolysis/gluconeogenesis represented the main metabolic pathways between PL5 *vs.* PL10. We randomly selected 19 DEGs to perform the qPCR analysis in order to verify the accuracy of RNA-Seq. According to the qPCR analysis (Figure 4), the expression patterns of these 19 DEGs were consistent with those of RNA-Seq, indicating the accuracy of the RNA-Seq.

### Identification of Gonad Differentiation and Development-Related Genes

A total of 29 genes were considered as the candidate genes involved in the mechanism of gonad differentiation and development of *M. nipponense* based on gene annotation and the differentially expressed patterns. A total of 19 genes were reported to be sex-related genes in vertebrates and crustaceans (Table 2), including insulin-like androgenic gland hormone factor (*IAG*), *DMRT11E*, transformer-2 (*Tra-2*), and double-sex (*DSX*), and 10 genes, which showed significant differences in at least two comparisons, including the juvenile hormone epoxide hydrolase (*JHEH*), dihydropyrimidinase (*DHP*), RNA and export factor–binding proteins (*ALY*), and skeletal muscle actin protein 6 (*SMA6*) (Table 3). The functions of these genes need to be investigated further.

**TABLE 2:** Identification of important sex-related genes in the transcriptome.

Unigene	E-value	Accession number	Species
Insulin-like androgenic gland factor	0	AGB56976.1	*Macrobrachium nipponense*
Sex-lethal 3	0	APO14322.1	*Macrobrachium resenbergii*
Transformer-2	3.37E-48	ACD13597.1	*Penaeus monodon*
Extra sex comb	0	AGI50961.1	*Macrobrachium nipponense*
Fushi-tarazu factor-1	1.61E-88	AAD41899.1	*Metapenaeus ensis*
GATA	5.28E-48	AUS76950.1	*Eriocheir sinensis*
Argonaute 2	5.52E-179	KC800811.1	*Penaeus monodon*
Argonaute 3	3.26E-157	AE014296.5	*Drosophila melanogaster*
Cytochrome p450 V20	5.65E-153	AFA26603.1	*Macrobrachium nipponense*
Cathepsin A	2.58E-134	AKO90271.1	*Tigriopus japonicus*
Cathepsin B	8.97E-124	AUG69383.1	*Macrobrachium rosenbergii*
Cathepsin D	8.02E-162	*ACG70181.1*	*Homarus americanus*
Cathepsin L	7.24E-109	AEC22811.1	*Macrobrachium nipponense*
Cyclin A	1.17E-80	AGG40745.1	*Litopenaeus vannamei*
Cyclin B	2.21E-125	ADB44902.1	*Macrobrachium nipponense*
Cyclin D	3.45E-09	EU939758.1	*Saccoglossus kowalevskii*
Ferritin	0	KC825355	*Macrobrachium nipponense*
Ferritin light-chain subunit	2.34E-28	ACR43472.1	*Rimicaris exoculata*
Dmrt11E	5.82E-92	AE014298.5	*Macrobrachium rosenbergii*

**TABLE 3:** Identification of important differentially expressed genes in the transcriptome.

Gene name	Accession number	E-value	Fold change (RNA-Seq)			
			PL5 vs. PL10	PL10 vs. PL15	PL15 vs. PL25M	PL15 vs. PL25F
Hemocyanin subunit 1	AGA17871.1	2.80E-64	11.56	11.29	4.45	7.93
Chitinase 4	AHL28109.1	0	4.35	5.91		
Hemoglobin	ABY61829.1	3.10E-26	−3.36	5.16		
*JHEH*	AKL71620.1	1.40E-185	−3.85	−4.25		
Hemocyanin	AHJ90473.1	3.40E-278	−4.19	−4.42	6.63	7.30
ALY	XP_024349462.1	6.40E-17			4.48	4.81
Dihydropyrimidinase	XP_018019690.1	4.70E-250	−5.57	−4.98		
Ubiquitin-2	BT036927.1	5.90E-48	−8.07	−6.03		
SMA6	FJ217212.1	1.20E-67		−6.62	−7.45	−6.6.2
Kynurenine formamidase	XP_018025634.1	3.00E-78		−7.34	7.06	

### The qPCR Analysis of Four DEGs in Different Developmental Stages

**FIGURE 5:**
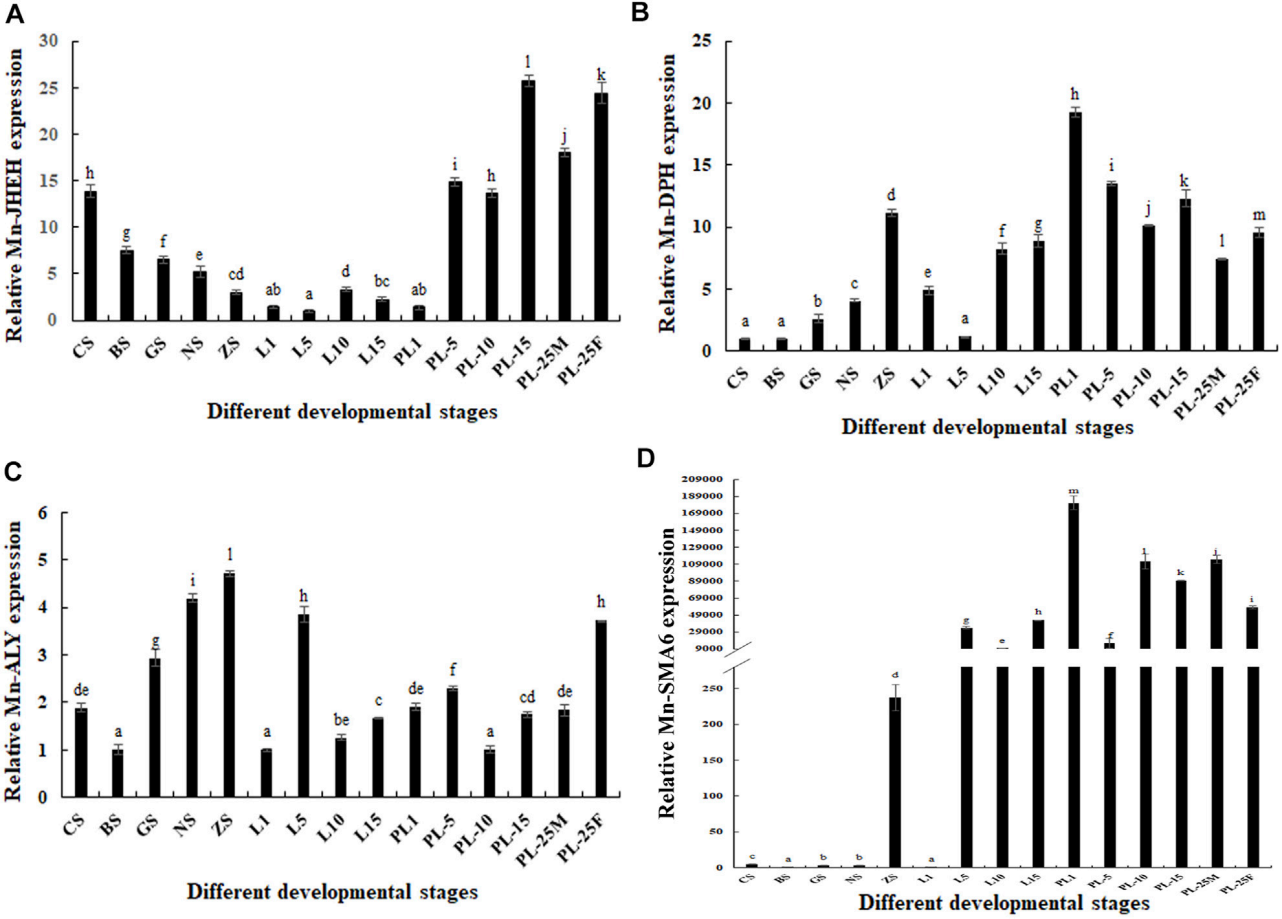
Expression analysis of four differentially expressed genes (DEGs) during the whole developmental stages. The amount of gene expressions was normalized to the *EIF* transcript level. Data are shown as mean ± standard deviation of tissues in three separate individuals. Lowercase indicates the expression difference (*p* < 0.05). **(A)** Characterization of the expression of *Mn-JHEH*; **(B)** characterization of the expression of *Mn-DHP*; **(C)** characterization of the expression of *Mn-ALY*; **(D)** characterization of the expression of *Mn-SMA6*.

*Mn-JHEH* was expressed at all tested developmental stages and showed significantly difference (*p* < 0.05). During the embryonic developmental stages, the highest developmental stage of *Mn-JHEH* was observed in the cleavage stage (*p* < 0.05) and gradually decreased with cell differentiation. During the larval developmental stages, the expressions of *Mn-JHEH* were generally lower than those of the embryonic developmental stages and post–larval developmental stages and showed the highest expression levels in L10 (larval developmental stage, L10). During the post–larval developmental stages, *Mn-JHEH* showed a higher expression than in the embryonic developmental stages and larval developmental stages and was higher in PL25F than was in PL25M. The highest expression level of *Mn-JHEH* during the whole developmental stages was observed in PL15, followed by PL25F, which was 25.47- and 23.79-fold higher than that observed in L5 (*p* < 0.05) (Figure 5A).

The expression levels of *Mn-DHP* during the embryonic developmental stages were gradually increased from the cleavage to zoea stage and showed significant difference (*p* < 0.05). During the larval developmental stages, the expression levels of *Mn-DHP* were gradually increased from L5 to L15. During the post–larval developmental stages, *Mn-DHP* reached the peak at PL1, and the expression at PL25F was 1.34-fold higher than that at PL25M (*p* < 0.05). The expressions of *Mn-DHP* during the post–larval developmental stages were generally higher than those during the embryonic developmental stages and larval developmental stages. The highest expression of *Mn-DHP* during the whole developmental process was observed at PL1, which was 19.13-fold higher than that at the cleavage stage (*p* < 0.05) (Figure 5B).

The lowest expression of *Mn-ALY* during the embryonic developmental stages was observed at the blastula stage and then gradually increased with cell differentiation and reached the peak at the zoea stage (*p* < 0.05). The *Mn-ALY* expression reached the peak at L5 during the larval developmental stages and showed significant difference with the other tested stages of larval development (*p* < 0.05). During the post–larval developmental stages, the lowest expression of *Mn-ALY* was observed at PL10 and then gradually increased with the development of juvenile prawn. The expression at PL25F was about two-fold higher than that at PL25M (*p* < 0.05). The highest expression of *Mn-ALY* during the whole developmental process was observed at the zoea stage, which was 4.63-fold higher than that at the blastula stage (*p* < 0.05) (Figure 5C).

During the embryonic developmental stages, the expressions of *Mn-SMA6* remained at a low level from the cleavage to nauplius stage and then sharply increased at the zoea stage, which was 231.29-fold higher than that at the blastula stage (*p* < 0.05). During the larval developmental stages, the lowest expression level of *Mn-SMA6* was observed at L1, and then significantly increased at L15 (*p* < 0.05). During the post–larval developmental stages, the highest expression of *Mn-SMA6* was observed at PL1, which showed significant difference with the other tested stages (*p* < 0.05). The expression at PL25M was about 1.5-fold higher than that at PL25M, which showed significant difference (*p* < 0.05) (Figure 5D).

### The Relative RNA Expression of *Mn-SMA6* in Different Mature Tissues

**FIGURE 6:**
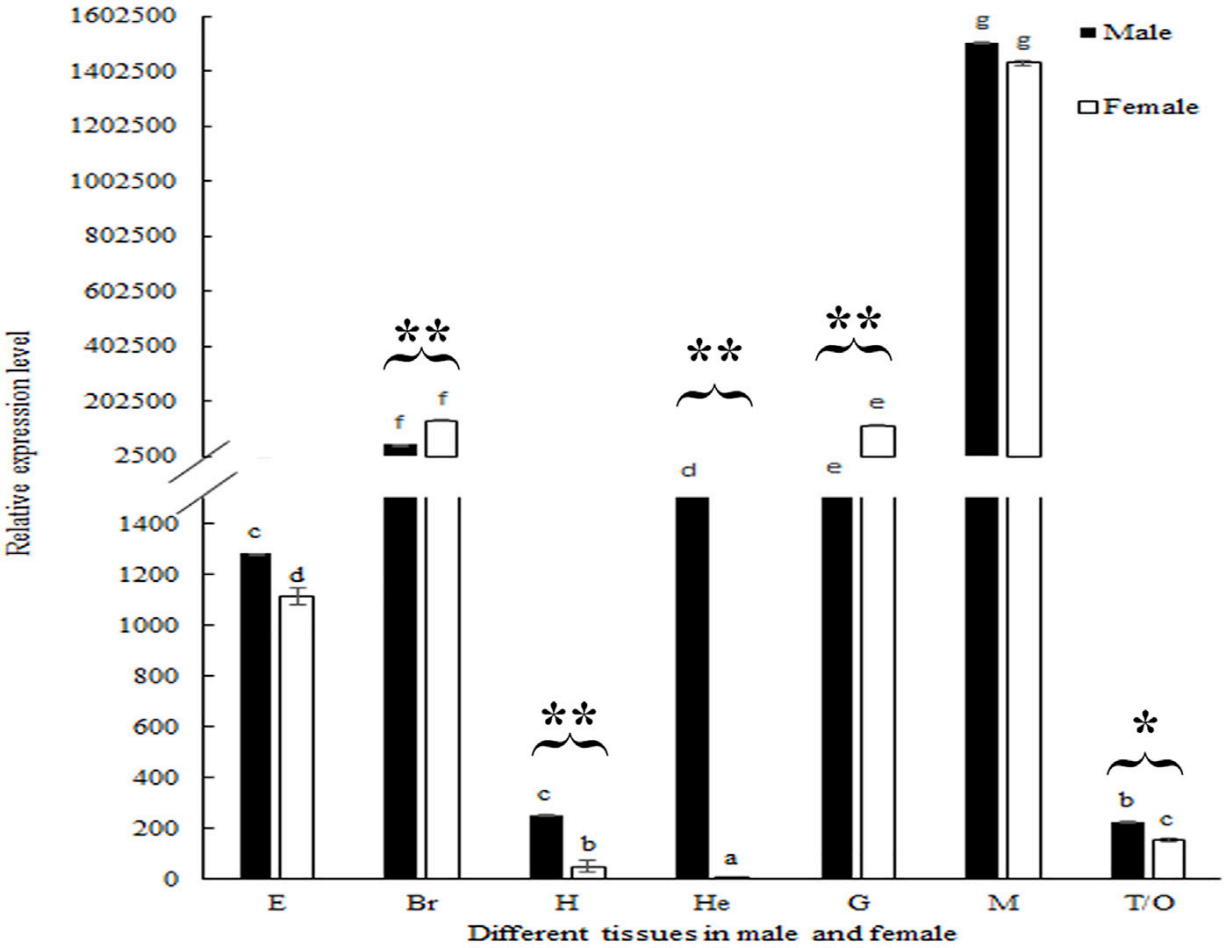
Expression analysis of *Mn-SMA6* in different mature tissues in male and female prawns. The amount of *Mn-SMA6* mRNA was normalized to the EIF transcript level. Data are shown as mean ± standard deviation of tissues from three separate individuals. Lowercase indicates the expression difference between different tissues of male and female prawns. * and ** indicate *p* < 0.05 and *p* < 0.01 in the same tissues between male and female prawns.

The biological functions of *Mn-SMA6* were further analyzed in different mature tissues of the male and female prawns. According to Figure 6, the highest expression levels of *Mn-SMA6* were observed in the muscles of both the male and female prawns, which showed significant difference with the other tested tissues (*p* < 0.05). *Mn-SMA6* showed higher expression levels in the eyestalk, heart, hepatopancreas, muscles, and gonads of the male prawns than those of the female prawns, while it showed higher expressions in the brain and gills of the female prawns. The expressions of *Mn-SMA6* showed significant differences in the brain, heart, hepatopancreas, gills, and gonads between the male and female prawns (*p* < 0.05).

### RNAi Analysis

**FIGURE 7:**
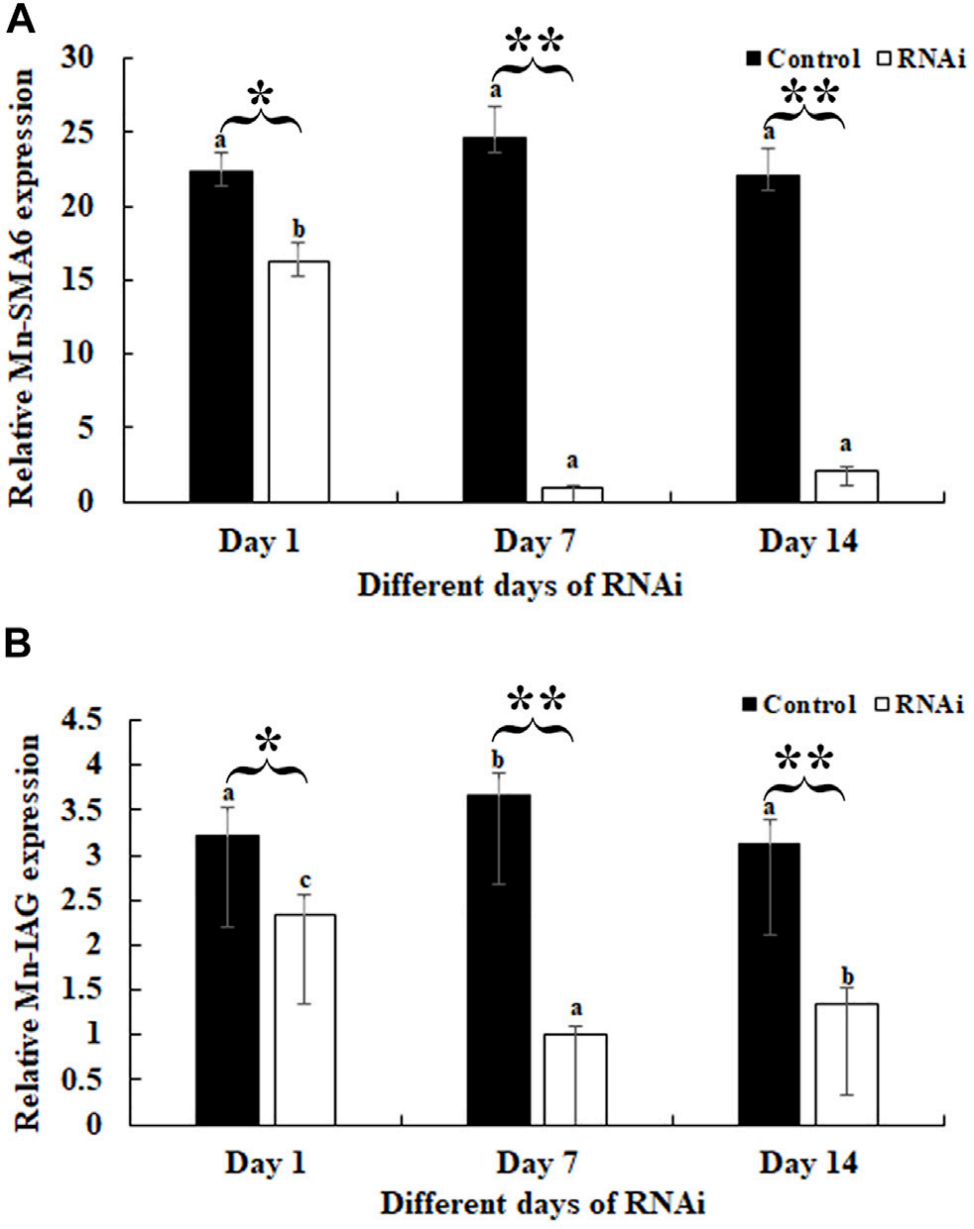
Expression analysis of *Mn-SMA6* and *Mn-IAG* after *Mn-SMA6* dsRNA injection. The amount of *Mn-SMA6* and *Mn-IAG* mRNA were normalized to the EIF transcript level. Data are shown as mean ± standard deviation of tissues from three separate individuals. Lowercase indicates the expression difference between the different days in the control group and RNAi group. * and ** indicate *p* < 0.05 and *p* < 0.01 between the RNAi group and control group on the sample day, respectively. **(A)** Expression analysis of *Mn-SMA6* after *Mn-SMA6* dsRNA injection. **(B)** Expression analysis of *Mn-IAG* after *Mn-SMA6* dsRNA injection.

RNAi was used to analyze the potential roles of *Mn-SMA6* in the sexual development of male prawns. The expressions of *Mn-SMA6* were determined by using the cDNA templates of the androgenic gland after the injection of *Mn-SMA6* dsRNA. The expressions of *Mn-SMA6* remained at a stable level in the control group and showed no significant difference between different days (*p* > 0.05). The expressions of *Mn-SMA6* significantly decreased at Days 7 and 14 after the injection of *Mn-SMA6* dsRNA. The decrease in *Mn-SMA6* expressions reached to 95 and 90% on Days 7 and 14, respectively, when compared with those in the control group on the same days (*p* < 0.01) (Figure 7A).

The expressions of *Mn-IAG* were also determined using the cDNA templates after the *Mn-SMA6* dsRNA injection. According to Figure 7B, the knockdown of the expression of *Mn-SMA6* showed positive regulatory relationship with that of *Mn-IAG*. The expressions of *Mn-IAG* were decreased with the decrease in expression of *Mn-SMA6*. The expression of *Mn-IAG* decreased to 73 and 58% on Days 7 and 14, respectively, when compared with that in the control group on the same days (*p* < 0.01).

### Histological Observations of Testis After RNAi

**FIGURE 8:**
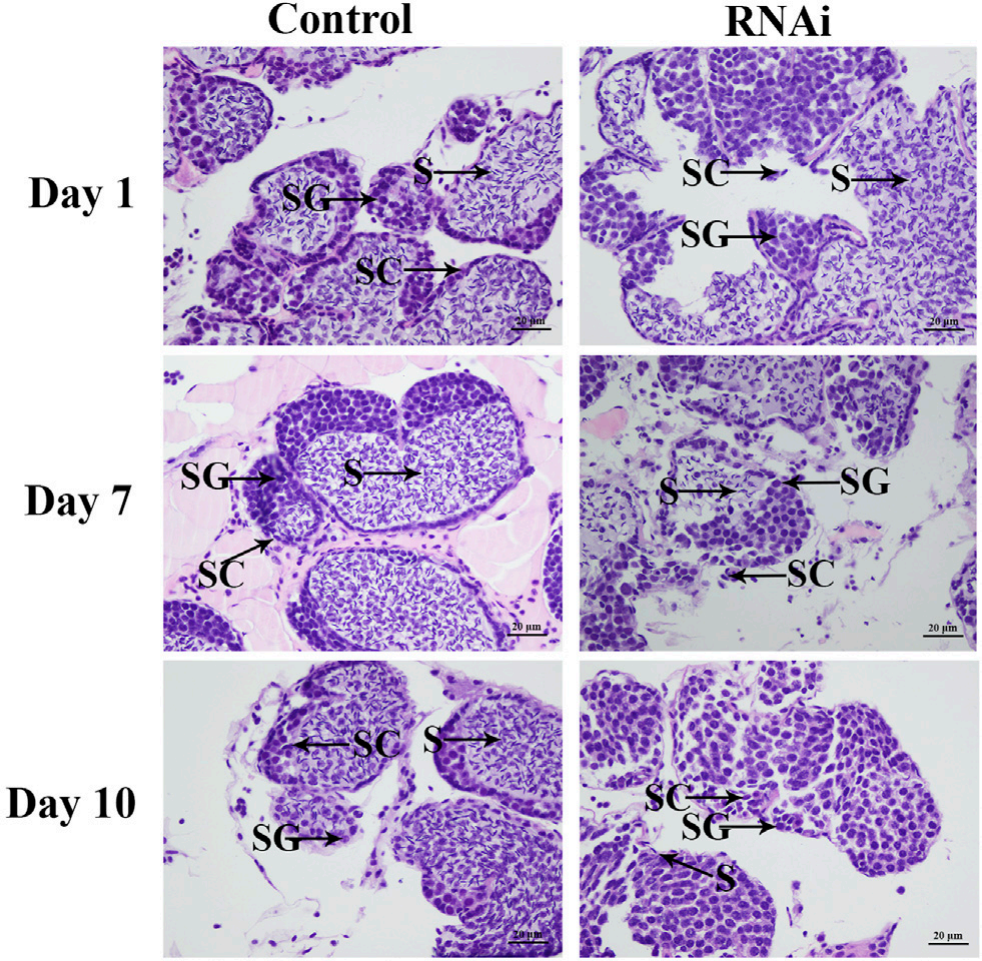
The histological observations of testis between the RNAi and control groups. SG: spermatogonia; SC: spermatocyte; S: sperm. Scale bars = 20 μm.

The testis development after the injection of *Mn-SMA6* dsRNA was determined by using HE staining. HE staining analysis showed that over 60% of the cell types were sperms in the control group, which were dramatically more than the number of spermatogonia and spermatocytes. The morphology of the cells did not differ significantly over time in the control group (Figure 8). However, the cell types showed significant difference between the different days in the RNAi group. The number of sperms decreased to only 35% on Day 7 after the injection of *Mn-SMA6* dsRNA. The sperms accounted for less than 3% of the cells on Day 14 after the injection of *Mn-SMA6* dsRNA (Figure 8).

## Discussion

The mechanism of sexual differentiation and development in crustacean species is a complicated biological process, which has received great attention in recent years. To the best of our knowledge, *IAG* was the most important gene, which was reported to positively regulate the male sexual differentiation and testis development in many crustacean species (Li et al., 2012; Ventura et al., 2012; Huang et al., 2014; Li et al., 2015; Liu et al., 2021; Zhou et al., 2021). However, the mechanism of gonad differentiation and development in crustaceans. In this study, the gonad differentiation and development sensitive period was identified by histological observation, and the important metabolic pathways and genes, which may be involved in the process of gonad differentiation and development in *M. nipponense*, were also selected through the transcriptome analysis. This study provides important evidence for the studies on gonad differentiation and development in crustaceans.

The histological observations revealed that the gonad primordium was not observed at PL5 in the juvenile prawns of *M. nipponense*, while it is observed at PL10. The gonad was differentiated at PL15, and the spermatogonia and oogonia were observed at this period. The gonads can be visually distinguished for the first time at PL25. During this period, the yolk was accumulated in the ovaries, and the sperms were observed in testes. The testis and ovary mature at this period. Thus, the gonad differentiation and development sensitive period was from PL5 to PL25 in *M. nipponense*. Compared with the sex sensitive period of other aquatic species (Song et al., 2006; Chen et al., 2007; Dai, 2010; You et al., 2012; You et al., 2016), the process of gonad development was dramatically fast. Thus, the establishment of an artificial technique to regulate the process of gonad development at the early stages is urgently required. The identification of the gonad differentiation and development sensitive period dramatically promote the studies to identify sex-related genes in *M. nipponense*. The regulation of the expressions of sex-related genes during this period plays vital roles in establishing the technique to regulate the process of gonad differentiation and development in *M. nipponense*.

In this study, a total of 49,103 unigenes were assembled, of which 18,430 unigenes were annotated. The genes participated in the mechanism of gonad differentiation and development and were predicted to be found in cell, cell part, cellular process, binding, and organelle in the GO assignment and the functional groups of general function prediction only, signal transduction mechanisms, posttranslational modification, protein turnover, and chaperones in the COG classification (Figure 3). This finding is consistent with previous studies (Jin et al., 2013; Jin^B^ et al., 2021). A total of 482, 98, 240, and 208 DEGs were identified between PL5 *vs.* PL10, PL10 *vs.* PL15, PL15 *vs.* PL25F, and PL15 *vs.* PL25M, respectively. The phagosome, lysosome, oxidative phosphorylation, and glycolysis/gluconeogenesis were the main enriched metabolic pathways between PL10 *vs.* PL15, PL15 *vs.* PL25F, and PL15 *vs.* PL25M. The gonad was identified to be differentiated and developed from PL10 to PL25. Many previous studies reported that immune-related and energy-related metabolic pathways played essential roles in the gonad development in *M. nipponense* (Jin et al., 2020; Jin^B^ et al., 2021), which is consistent with this study. In addition, a total of 29 genes were considered as the strong candidate genes, involved in the mechanism of gonad differentiation and development of *M. nipponense*, based on gene annotation and differentially expressed pattern.

The phagosome and lysosome were the main enriched metabolic pathways between PL10 vs PL15, and the most enriched metabolic pathways between PL15 vs PL25F and PL15 vs PL25M. The lysosomes function in decomposing nucleic acids, proteins, polysaccharides, and other biological macromolecules. The lysosomes contain many hydrolases. These hydrolases decompose the substances those enter into the cells from the outside or digest the local cytoplasm or organelles in the cells. The aged cells will be digested by the hydrolases released from the lysosomes (Duve and Wattiaux, 1966; Luzio et al., 2007). Phagocytosis plays essential roles in tissue remodeling, inflammation, and defense against infectious agents, which is the process by which large particles are absorbed by a cell. A phagosome is formed through a combination of specific receptors on the phagocyte surface and ligands on the particle surface. Most bacteria are killed and degraded into fragments by toxic products released through the fusion of phagosomes and lysosomes. A reasonable explanation for this is that cell differentiation is vigorous during this period. Thus, the aged or redundant cells need to be digested in order to maintain the normal gonad development.

Adenosine triphosphate (ATP) is a high-energy compound, which is used as an energy source in nearly all metabolic activities, including the gonad differentiation and development. Interestingly, oxidative phosphorylation and glycolysis/gluconeogenesis were the main enriched metabolic pathways from PL10 to PL25. Both oxidative phosphorylation and glycolysis/gluconeogenesis are energy metabolism–related metabolic pathways. Oxidative phosphorylation occurs in the cytoplasm of prokaryotes or in the inner membrane of the mitochondria of eukaryotic cells. The energy released from the oxidation of substances *in vivo*, which has positive effects on the coupling reaction between inorganic phosphate and adenosine diphosphate. ATP is synthesized through the respiratory chain (Dimroth et al., 2000). Glycolysis/gluconeogenesis has positive effects on the production of pyruvate (CH_3_COCOO^−^ + H^+^) from glucose (C_6_H_12_O_6_). Free energy is released to form ATP and reduced nicotinamide adenine dinucleotide (Lubert, 1995). A reasonable explanation for this is that oxidative phosphorylation and glycolysis/gluconeogenesis provide the energy for the early stages of gonad differentiation and development in juvenile prawns of *M. nipponense*.

A total of four novel genes were selected as the strong novel candidate genes, which were involved in the mechanism of gonad differentiation and development in *M. nipponense*. These novel genes were selected based on the changes of gene expression during the gonad differentiation and development sensitive period and previously functional analysis. Juvenile hormone (*JH*) was reported to have positive regulatory roles on the process of molting, metamorphosis, reproduction, diapause, and reproduction in insects (Mackert et al., 2010; Lü et al., 2015; Zhu et al., 2017). The level of *JH* keeps the balance in insects through the synthesis and degradation of *JH*. *JHEH* has dramatic effects on the degradation of *JH*, which can convert *JH* into the juvenile hormone Diol (Xu et al., 2014; Lü et al., 2015). Thus, *JHEH* plays essential roles in the process of growth, development, and reproduction in insects. During the whole developmental process of *M. nipponense*, the *Mn-JHEH* showed a higher expression level during the gonad differentiation and development sensitive period than during the other tested stages, indicating that *JHEH* promotes the process of gonad differentiation and development in *M. nipponense*. In addition, the *Mn-JHEH* expression in female prawns at PL25 was higher than in male prawns at the same stage, indicating that *JHEH* plays more essential roles in female sexual development.

*DHP* is a zinc metalloenzyme, which can degrade dihydropyrimidine. In vertebrates, the accumulation of *DHP* has negative effects on DNA replication and transcription (Basbous et al., 2020). In *Xenopus laevis*, dihydropyrimidine directly interferes with the recombination of plasmid and chromosomal DNA (Basbous et al., 2020). In mice, *in situ* hybridization analysis showed that *DHP* was localized in the sperms after meiosis (Kato et al., 1998). To the best of our knowledge, the research on *DHP* mainly focuses on vertebrates, and no report was found in crustaceans. The highest expression levels of *Mn-DHP* during embryonic developmental stages and larval developmental stages were observed at the zoea stage and at L1, respectively, indicating that it promotes hatching and the metamorphosis process in *M. nipponense* (Zhang et al., 2013). The expression levels of *Mn-DHP* during gonad differentiation and development sensitive period were generally higher than those during the embryonic developmental process and larval developmental stages, indicating that it plays positive regulatory roles in gonad differentiation and development of *M. nipponense*. As the same as that of *Mn-JHEH* expression, *Mn-DHP* showed a higher expression in the female prawns at PL25 than in male prawns at PL25 (*p* < 0.05), indicating that *DHP* plays more essential roles in the female sexual development in *M. nipponense*.

The *ALY* gene is highly conserved between different species. It has an RNA binding domain, which can bind to the RNA and Tap, playing essential roles in the connection between RNA and Tap. In yeast, an increase in concentration of *YrALY* can enhance the transport of mRNA, while inhibiting the expression of *YrALY* has negative effects on the transport of mRNA (Strasser and Hurt, 2000). *ALY* was reported to be a limiting factor in mRNA transport in *X. laevis* (Zhou et al., 2000; Luo et al., 2001; Rodrigues et al., 2001). The expressions of *Mn-ALY* were gradually increased from the blastula stage to zoea stage during the embryonic developmental stages, indicating that *ALY* plays essential roles in cell differentiation. The expressions of *Mn-ALY* during the embryonic developmental stages were slightly higher than in the larval developmental stages and post–larval developmental stages. *Mn-ALY* expressions showed sexual differentiation between males and females. *Mn-ALY* expression in female prawns was almost two-fold higher than in male prawns, indicating that *ALY* plays more essential roles in female sexual development.

*SMA6* is the main component of the myofibrils of contractile muscle cells and the main cytoskeleton component of non–muscle cells. *SMA6* is a specific protein in male *Drosophila*, playing essential roles in the formation of male-specific muscles and the development of its special characteristics (Currie et al., 1995). The expression of *Mn-SMA6* was significantly increased at the zoea stage, indicating that *SMA6* was involved in the process of hatching in *M. nipponense*. In addition, the *Mn-SMA6* expression during the larval and post–larval developmental stages remained at a high level, indicating that *SMA6* has multiple functions during the development of *M. nipponense*, including the gonad differentiation and development. The *Mn-SMA6* expression in male prawns at PL25 was higher than that in female prawns, indicating that *SMA6* may play more essential roles in male sexual development in *M. nipponense*.

The qPCR analysis of *Mn-SMA6* in different mature tissues showed that *SMA6* generally showed higher expression levels in male prawns than in female prawns in the same tissues. This indicates that *SMA6* plays more essential roles in male sexual development, which is consistent with the prediction of qPCR analysis during the different developmental stages. The expressions of *Mn-SMA6* were significantly decreased at Days 7 and 14 after the injection of *Mn-SMA6* dsRNA, indicating that the synthesized *Mn-SMA6* dsRNA was efficient in knocking down the expression of *SMA6* in *M. nipponense*. Interestingly, the expressions of *Mn-IAG* were also decreased with a decrease in *Mn-SMA6* expression, which shows the positive regulatory relationship between *SMA6* and *IAG* in *M. nipponense*. *IAG* is an important hormone that is secreted by the androgenic gland. It was reported to be the most important sex-related gene in the crustacean species, playing essential roles in promoting male sexual differentiation and development (Ventura et al., 2009; Rosen et al., 2010; Ventura et al., 2011). Knocking down the expression of *IAG* resulted in sex-reversal in *Macrobrachium resenbergii* (Ventura et al., 2012). Thus, the positive regulatory roles between *SMA6* and *IAG* also suggested that *SMA6* was involved in the male sexual development in *M. nipponense*. Histological observations of the testes after *Mn-SMA6* dsRNA injection revealed that the sperms were rarely formed at Day 14 after the injection of *Mn-SMA6* dsRNA, indicating the positive regulatory roles of *SMA6* on testes development in *M. nipponense*.

In conclusion, the sensitive period of gonad differentiation was from PL10 to PL25 in *M. nipponense*, determined by HE staining. A total of 29 genes were predicted to play essential roles in the process of gonad differentiation and development of *M. nipponense* through the transcriptome profiling analysis during the gonad differentiation and development sensitive period based on the gene annotation and gene expression pattern. Phagosome, lysosome, oxidative phosphorylation, and glycolysis/gluconeogenesis were predicted to play essential roles in the mechanism of gonad differentiation and development in *M. nipponense*. qPCR analysis of four DEGs during the whole developmental stages revealed that these four DEGs were highly expressed during the sensitive period of gonad differentiation and development in *M. nipponense*. *JHEH*, *DHP*, and *ALY* were potentially involved in female sexual development in *M. nipponense*, while *SMA6* plays more essential roles in the male sexual development. Further functional analysis of *Mn-SMA6* by RNAi revealed that *SMA6* positively regulated the expression of *Mn-IAG* and testis development in *M. nipponense*. This study provided valuable evidences on the regulation of gonad development in *M. nipponense*, maintaining the sustainable development of *M. nipponense* industry. We also confirmed that *SMA6* positively regulated the testis development in *M. nipponense* by using RNAi, combined with histological observations.

## Data Availability

The data sets presented in this study can be found in online repositories. The names of the repository/repositories and accession number(s) can be found in the article/supplementary material.
